# A hypothesis for the characteristic parafoveal toxicity observed in hydroxychloroquine retinopathy

**DOI:** 10.1038/s41433-026-04270-1

**Published:** 2026-01-31

**Authors:** Michele Chan, Nigel Davies

**Affiliations:** Guy’s and St Thomas’ NHS Foundation Trust, London, UK

**Keywords:** Pathogenesis, Anatomy

Hydroxychloroquine (HCQ) retinopathy is characterised by a classical pattern of concentric parafoveal retinal pigment epithelium (RPE) loss, resulting in a corresponding ring-like visual field defect. Despite well-documented evidence of the distinctive spatial distribution and dose-dependent risk factors, the pathophysiological mechanism underlying the selective vulnerability of the parafoveal region remains undefined [1]. In the context of recent revisions in the screening and diagnostic criteria of HCQ retinopathy by the Royal College of Ophthalmologists [2], we propose a novel hypothesis for the characteristic toxicity pattern observed in HCQ retinopathy, thereby establishing a theoretical foundation for future research directions and population-based studies.

One proposed theory of retinopathy at present points to inhibition of the recycling or uptake of all-trans-retinol in the RPE by HCQ [3] however, it is not clear why parafoveal photoreceptors are particularly susceptible to damage. Another suggested mechanism attributes the melanin-binding effect of HCQ [4] to the damage of melanin-containing ocular tissues. Interestingly, the choroidal melanin occupancy rate of the centre ring was shown to be significantly larger than the outer ring within a 5-mm circular region from the foveal centre in healthy subjects [5].

Macular pigment (MP) has generated much research interest over the last decade. Composed of carotenoids meso-zeaxanthin (MZ), lutein (L) and zeaxanthin (Z), MP is a yellow pigment found in the highest concentrations in Henle’s fibre layer at the fovea and the inner nuclear layer in the parafovea. In particular, L is the dominant carotenoid stereoisomer in the outer macula, Z in the mid-peripheral macula and MZ at the inner macula [6].

MP possess several important properties that might prevent oxidative injury at the macula by absorbing blue light (peak 460 nm) and eliminating reactive oxygen species (ROS), supporting visual function. Lower levels of MP have been associated with retinal diseases, including diabetic retinopathy, retinopathy of prematurity, age-related macular degeneration (AMD) and Alzheimer’s Disease [7–9]. In turn, oral supplementation of L, Z and MZ has been shown to enhance visual performance in both healthy and diseased retinas [10].

We propose that the spatial relationship between melanin and MP concentrations in the macula could contribute to the distinctive parafoveal distribution of disease observed in HCQ retinopathy.

**Fig. 1:**
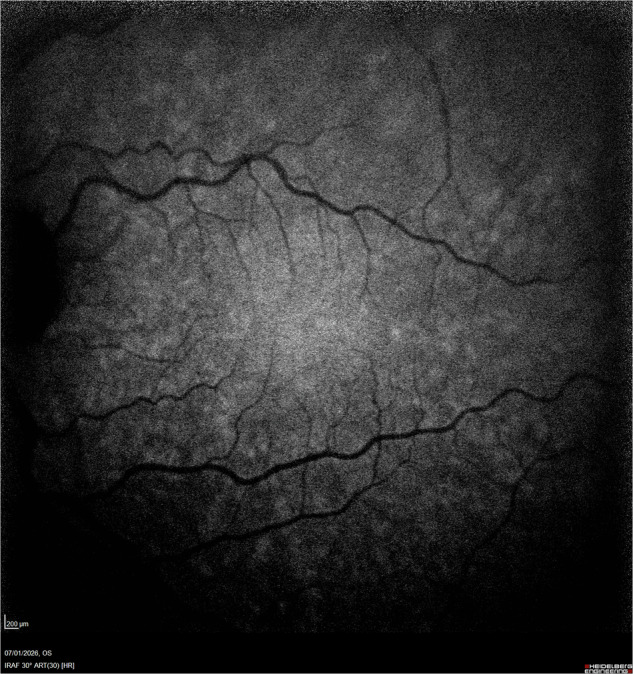
Infrared autofluorescence (IR-AF) imaging of a healthy eye, indicative of melanin concentration.

**Fig. 2:**
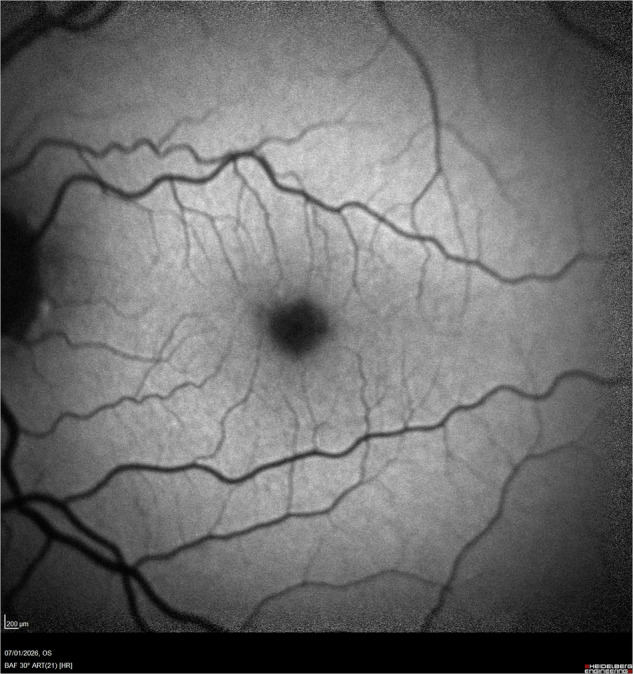
Blue autofluorescence (AF) imaging of the same eye, indicative of macular pigment concentration.

While HCQ is known to preferentially accumulate in melanin-rich ocular tissues, including the RPE, we hypothesise that MPs provide photoprotective and antioxidative effects that mitigate HCQ-induced cellular damage: the central fovea region is therefore spared from toxicity due to high concentration of MPs. Conversely, in the parafoveal region, melanin concentrations remain relatively high while MP density sharply declines (estimated 100-fold decrease from the foveal center to peripheral retina) [11], rendering this region more susceptible to HCQ-induced toxicity. A comparison of infrared with blue autofluorescence images in a healthy eye demonstrates that melanin concentration (Fig. 1) in the macula goes beyond MP concentration (Fig. 2).

With recent updates to guidelines from the Royal College of Ophthalmology for the screening and diagnostic criteria for HCQ Retinopathy in 2020 [2], our proposed mechanistic hypothesis offers important clinical implications. Firstly, it reinforces the rational for spectral-domain optical coherence tomography and fundus autofluorescence imaging for screening of patients at risk of developing retinopathy, as these modalities are well-suited to detect parafoveal RPE changes where early disease typically manifests and note the central ring-like field defect too. Secondly, it suggests that individual variation in MP optical density (MPOD) may represent a previously unrecognised risk modifier. Patients with constitutively low MPOD due to age, genetic predisposition or dietary factors may be more susceptible to drug-induced toxicity in the parafoveal region.

In a clinic setting, this raises a simple and achievable strategy for primary prevention of HCQ-induced retinopathy. If MP depletion in the parafoveal region indeed plays a role in the development of toxicity, prophylactic supplementation with MP constituents in patients initiating HCQ may be warranted. Lutein and zeaxanthin supplementation is shown to increase MPOD [12, 13] and are widely available, cost-effective, with established safety profiles [14, 15].

Here we present several potential directions to guide future investigations. However, we acknowledge that the design of prospective studies poses significant practical challenges: HCQ retinopathy typically develops after at least five years of cumulative exposure, with the highest risk after more than 20 years of use [16]. This extended disease course necessitates long follow-up periods and substantial resources for adequately powered randomised controlled trials of preventive interventions.

Nevertheless, alternative study designs can provide meaningful evidence within more feasible timeframes. Retrospective cohort studies examining baseline MPOD in patients who subsequently developed HCQ retinopathy could provide insight into disease susceptibility. Cross-sectional studies correlating the spatial extent of retinal damage with individual MPOD profiles could explore whether the boundary of toxicity corresponds to the parafoveal zone of MP depletion.

At the cellular level, in vitro studies could evaluate whether lutein and zeaxanthin supplementation reduce HCQ-induced cytotoxicity. Animal models of HCQ retinopathy, particularly in species with MPOD profiles similar to human eyes, could investigate the role of modulating MP levels in the development of toxicity under accelerated exposure.

Importantly, given the established safety profile of lutein and zeaxanthin supplementation and the prospect of irreversible visual field loss associated with HCQ retinopathy, the threshold for clinical adoption may not require decade-long prospective trials. Convergent evidence from mechanistic studies, observational data and surrogate endpoint trials may provide sufficient justification for recommending MP supplementation as a low-risk adjunctive measure in patients requiring long-term HCQ therapy.
